# Stromal Collagen Arrangement Correlates with Stiffness of the Canine Cornea

**DOI:** 10.3390/bioengineering7010004

**Published:** 2019-12-25

**Authors:** Brian C. Leonard, Krista Cosert, Moritz Winkler, Ariana Marangakis, Sara M. Thomasy, Christopher J. Murphy, James V. Jester, Vijay Krishna Raghunathan

**Affiliations:** 1 Department of Surgical and Radiological Sciences, School of Veterinary Medicine, University of California, Davis, CA 95616, USA; bcleonard@ucdavis.edu (B.C.L.); kmcosert@gmail.com (K.C.); admarangakis@ucdavis.edu (A.M.); smthomasy@ucdavis.edu (S.M.T.); cjmurphy@ucdavis.edu (C.J.M.); 2 Gavin Herbert Eye Institute and the Department of Biomedical Engineering, University of California, Irvine, CA 92697, USA; moe2k5@gmail.com (M.W.); jjester@hs.uci.edu (J.V.J.); 3 Department of Ophthalmology & Vision Science, School of Medicine, University of California, Davis, CA 95616, USA; 4 The Ocular Surface Institute, College of Optometry, University of Houston, Houston, TX 77204, USA; 5 Department of Basic Sciences, College of Optometry, University of Houston, Houston, TX 77204, USA; 6 Department of Biomedical Engineering, Cullen College of Engineering, University of Houston, Houston, TX 77204, USA

**Keywords:** corneal stroma, collagen arrangement, second-harmonic generation imaging, atomic force microscopy, elastic modulus, canine

## Abstract

The cornea is the most external layer of the eye and serves two important roles in (1) the refraction of light and (2) protection from the outside environment, both of which are highly dependent on the collagen assembly of the corneal stroma. This study sought to determine the collagen fiber arrangement of the canine corneal stroma and correlate the stromal organization with tissue stiffness in the anterior and posterior cornea. Collagen organization of the canine cornea was visualized through second-harmonic generation (SHG) imaging, and tissue stiffness of the anterior and posterior corneal stroma was determined by atomic force microscopy. Analysis of the canine anterior corneal stroma using SHG imaging documented intertwining of the collagen fibers with a high degree of fiber branching, with a more lamellar and non-branching posterior stroma. The anterior stroma had significantly higher tissue stiffness in both dogs and humans, when compared with the posterior corneal stroma (canine median: 1.3 kPa vs. 0.3 kPa; human median: 14.6 kPa vs. 2.1 kPa, respectively). There was a direct correlation between corneal collagen stromal organization and tissue stiffness in the dog, which was consistent with other mammalian species previously examined and likely reflects the need for maintenance of rigidity and corneal curvature.

## 1. Introduction

The cornea is responsible for many functions of the eye, serving as the primary refractive interface for light entering the eye, as well as forming an anterior physical barrier to the external environment. The corneal stroma comprises nearly 90% of the total corneal thickness in most domesticated mammals, and is primarily composed of stromal collagen [1]. The individual collagen fibrils of the corneal stroma are arranged in larger bundles called fibers, which are further arranged into larger units called lamellae [2,3,4]. Importantly, the biomechanical strength of the cornea is thought to be directly related to the organization of these fibers and lamellae, however, the mechanisms by which they form these complex arrangements remain poorly understood.

Second-harmonic generation (SHG) imaging is a nonlinear optical imaging technique that allows for visualization of collagen fiber arrangement [3]. Briefly, SHG images are generated using ultrashort infrared laser pulses that result in the oscillating polarization of structures that lack central symmetry, such as collagen [5]. This induced polarization of the collagen fibrils yields light emissions from a very small focal volume that are exactly half the wavelength of the incident laser beam, and the detection of these light emissions permits the visualized reconstruction of the collagen fiber arrangement within the examined tissue [5]. The use of SHG imaging is ideal for characterizing corneal stromal tissue due to the primarily collagen composition of the stroma and the ability of infrared light to penetrate the entire thickness of the cornea, generating an image with high axial and lateral resolution [6]. A recent comparative study of the corneal stroma using SHG imaging demonstrated that there is a high degree of diversity in the three-dimensional branching of fibers within the corneal stroma, with birds having more branching than other nonmammalian vertebrates (reptiles, amphibians, fish) [7]. While there are differences among species in the extent of the interweaving of fibers, mammalian corneas generally exhibit intertwining collagen fibers of seemingly random orientation in the anterior corneal stroma with less interweaving of fibers found in the posterior stroma [7,8].

The organization of the collagen fibers of the corneal stroma has been shown to be directly correlated with corneal stromal stiffness. Using indentation techniques, such as atomic force microscopy (AFM)-based nanoindentation, it is possible to assess the stiffness of unfixed biologic tissue. In both the human and rabbit cornea, the anterior stroma has been shown to be stiffer than the posterior stroma, likely due to the differences noted in the degree of collagen fiber intertwining between the anterior and posterior stroma [6,8]. The combination of increased collagen fiber intertwining and increased tissue stiffness in the anterior stroma emphasizes the evolutionary pressure to maintain mechanical rigidity to support the two key roles of the cornea, refraction of light (dependent on maintenance of curvature) and protection from injury. This concept of tissue organization and stiffness is of particular relevance to the design and testing of bioartificial corneas whereby, despite being composed of collagen, the tensile mechanical properties of these synthetic tissues in a laboratory animal may not approximate that of the human [9,10,11]. Therefore, evaluating the structural and biomechanical properties of the canine cornea may aid in their use as a large animal model for bioartificial corneas.

The purpose of this study was to characterize the collagen fiber organization in the canine cornea utilizing SHG imaging and determine if stromal arrangement correlated with the tissue stiffness of the anterior and posterior corneal stroma. We hypothesized that the collagen fiber arrangement within the canine cornea would have similarities to other previously characterized mammals, and that the corneal stromal stiffness would differ based on the collagen fiber organization between the anterior and posterior stroma. 

## 2. Materials and Methods 

### 2.1. Canine Eyes

Canine globes (one globe for SHG imaging, two globes from separate individuals for atomic force microscopy) from seven-month-old purpose bred Beagles were procured from animals that were euthanized for reasons unrelated to the current study. Immediately after euthanasia, the eyes were enucleated and transported overnight chilled and wrapped in gauze soaked with phosphate buffer solution (PBS) for processing the following day for either SHG imaging or AFM. On examination, the globes were deemed free of corneal disease.

### 2.2. Human Corneal Buttons

Human corneal buttons (n = 2) were procured from Saving Sight (St. Louis, MO, USA) stored in Optisol (Bausch & Lomb, Rochester, NY, USA) at 4 °C and used at less than or at 3 weeks postmortem. The selected human corneal buttons lacked historical or observable corneal stromal pathology.

### 2.3. Second-Harmonic Generation Imaging

A single canine globe was fixed by perfusion under pressure using 4% paraformaldehyde in PBS (pH 7.4). The cornea was then dissected from the remainder of the globe and bisected mid-sagittally. One of the sections was vertically embedded in low melting point agarose (NuSieve GTG, Rockland, ME, USA) and a vibratome (Vibratome 1500, Intracel Ltd., Shepreth, UK) was used to cut multiple 300 μm thick sections. The cut sections were then stored in PBS until imaging. Cross-sectional imaging of the cornea was performed as previously described [6,8]. Briefly, the sections of canine cornea were scanned using a Zeiss 510 LSM Meta microscope (Carl Zeiss Inc., Thornwood, NY, USA) at 820 nm. Second-harmonic generated light was detected and several hundred consecutive overlapping images were acquired and stitched together using custom-coded ImageJ scripts. 

### 2.4. Atomic Force Microscopy

#### 2.4.1. Sample Preparation

For the canine globes, an 8 mm diameter corneal trephine was used to mark the central cornea and the corneal epithelium was removed using an excimer laser spatula (BD Vistec, Franklin Lakes, NJ, USA) within the marked region. An excimer laser (Nidek Excimer Laser Corneal Surgery System EC-5000, Fremont, CA, USA) was used to photoablate the epithelial basement membrane and stroma to expose either the very anterior corneal stroma (1 cycle of 6 mm diameter at 40 Hz with 83 pulses ablated at 50 μm depth) or the posterior corneal stroma (2 cycles of 6 mm diameter at 40 Hz with 292 pulses in each cycle ablated at 175 μm depth, i.e., a total of 350 μm total depth was ablated at the end of 2 cycles) in the center of the epithelial wound. The cornea was then carefully dissected from the remainder of the globe. Human corneal buttons that are unsuitable for transplant generally have fewer corneal endothelial cells resulting in edema. Thus, the excimer laser treatment depth was adjusted to factor for edema while attaining appropriate stromal depth. Subsequently, for the human corneal buttons, the corneal epithelium was removed using an excimer spatula and an excimer laser was used to photoablate the epithelial basement membrane and stroma to expose the anterior corneal stroma (1 cycle of 6 mm diameter, 40 Hz, 59 pulses, 20 μm depth) or the posterior corneal stroma (2 cycles 6 mm diameter, 40 Hz, 294 pulses, 100 μm depth; 1 cycle 6 mm diameter, 40 Hz, 59 pulses, 20 μm depth, i.e., 220 μm total depth), and the positioning within the stroma was confirmed using spectral domain optical coherence tomography (SD-OCT, RTVue 100, Optovue, Inc., Fremont, CA, USA) (anterior stroma: 5% into the stroma, posterior stroma: 85% into the stroma).

#### 2.4.2. AFM

Using the Soft-Clamping Immobilizing Retainer of Tissue (SCIRT) method, the cornea was placed into an AFM dish that had been coated with cured dielectric silicone (Sylgard 527, Dow Corning, Midland, MI, USA) with the anterior-most aspect of the tissue oriented upwards [12]. The SCIRT was placed on top of the tissue, which was further immobilized by using a ring of cyanoacrylate glue around the perimeter of the cornea. The sample was fully immersed in Dulbecco’s phosphate buffered saline, which helps to cure the cyanoacrylate glue while maintaining tissue hydration. Silicon nitride cantilevers (PNP-TR-50, Nano World, Neuchâtel, Switzerland) modified with a borosilicate bead (5 μm nominal radius) were used to generate five indentation force vs. indentation depth curves (MFP-3DBIO Asylum Research, Santa Barbara, CA, USA) in contact mode at 2 μm/s for each sample from at least 5 random locations around the central cornea. For the canine and human corneas, there were 48 and ~100 AFM measurements made in the anterior and posterior stroma, respectively. Moduli measurements were obtained by mathematically fitting the indentation curves to the Hertz model for spherical geometry as described previously [13].

### 2.5. Statistical Analysis

The D’Agostino-Pearson test was performed to assess the normality of the data, then a two-tailed Mann-Whitney ranked sum test was performed on the AFM measurements from the anterior and posterior corneal stroma using Prism 8 (GraphPad Software, La Jolla, CA, USA). Data was represented as box and whisker plots with the box extending to the 25th and 75th percentiles, with the median as the line in the middle of the box and whiskers representing the minimum and maximum values. In order to compare the elastic moduli obtained from canine corneas with those from human and rabbit tissues, the mean modulus measurements and standard deviation were calculated.

## 3. Results

### 3.1. Second-Harmonic Generation Imaging of Canine Corneal Stroma

**Figure 1:**
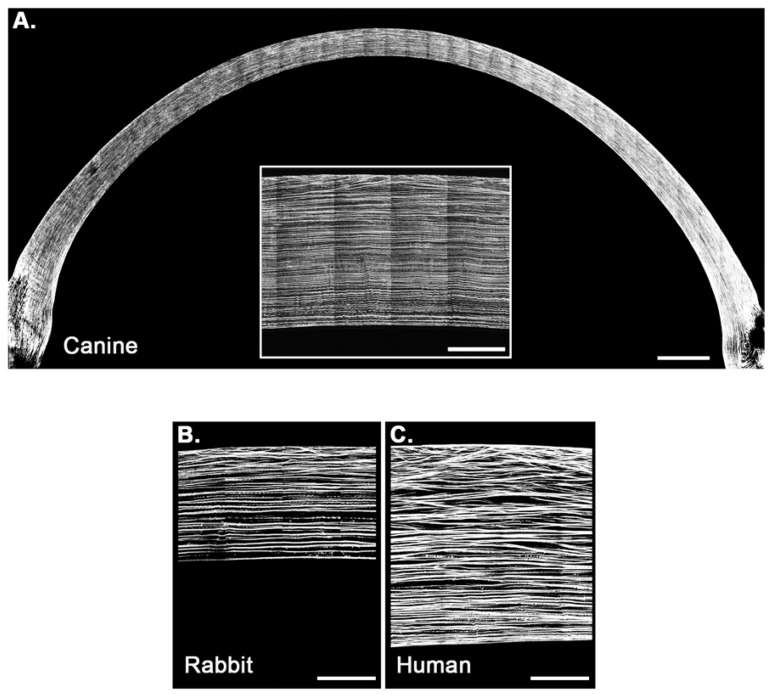
The collagen fiber arrangement of the canine cornea (**A**) is consistent with other mammals, with an intertwining pattern similar to that of the rabbit cornea (**B**) but with markedly less intertwining when compared with the human cornea (**C**) [8]. The anterior-most aspect (30%) of the canine cornea (**A**) exhibits a thin superficial region of intertwining collagen fibers whereas the posterior aspect (70%) exhibits a parallel collagen fiber arrangement, nearly identical to the rabbit (**B**). By contrast, the human cornea (**C**) exhibits extensive collagen fiber intertwining throughout the majority of the cornea and smaller area of parallel fiber arrangement in the posterior aspect. Scale bar equivalent to 1 mm (**A**), 200 μm (**A** inset, **B** and **C**). The faint vertical lines seen in the (**A** inset), **B**,**C** represent artifact from the image stitching process. Figures **B** and **C** are reprinted from *Acta Biomater.*, Vol. 10, Iss. 2, Thomasy SM et al., “Elastic modulus and collagen organization of the rabbit cornea: epithelium to endothelium”, pp. 785–791, Copyright (2014), with permission from Elsevier.

Using SHG imaging, the orientation and alignment of the corneal collagen fibers were visualized with the fixed specimen from limbus to limbus (Figure 1). The anterior stromal collagen fibers were highly interwoven, leading to branching and anastomosing with other collagen fibers in nonadjacent layers of the anterior 30% of the stroma (Figure 1 inset). In the posterior 70% of the stroma, the arrangement of the collagen fibers was markedly different, as demonstrated by the fibers being oriented in a parallel fashion and an absence of interweaving, branching or anastomosing with other surrounding collagen lamellae. 

### 3.2. Atomic Force Microscopy of Canine and Human Corneal Stroma

**Figure 2:**
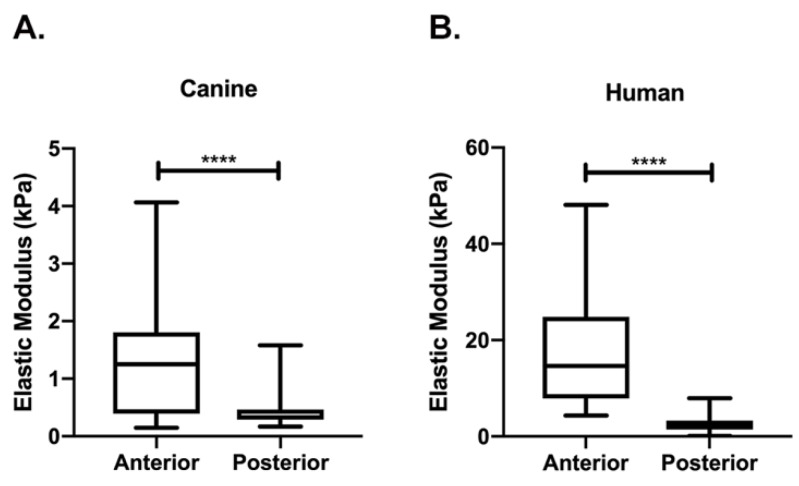
The anterior stroma of the cornea is significantly stiffer than the posterior corneal stroma in both the canine (**A**) and human (**B**). Box and whisker plots of elastic modulus (kPa = kilopascal) measured using atomic force microscopy (AFM) where the edges of the box represent the 25th and 75th percentile, the line within the box represents the median and whiskers extend to the maximum and minimum values. Box and whisker plots derived from the summation of individual AFM measurements of the anterior and posterior stroma from two canine (**A**) and two human (**B**) corneas. A Mann-Whitney test (nonparametric *t*-test) was performed, **** signifies *p* < 0.0001.

The median tissue stiffness of the canine anterior stroma (1.3 kPa) was significantly increased (nearly 4-fold) when compared with the posterior stroma (0.3 kPa) (*p* < 0.0001; Figure 2). Similarly, the human cornea has a significantly higher median anterior stromal tissue stiffness (14.6 kPa) when compared to the posterior stroma (2.1 kPa) (*p* < 0.0001; Figure 2) yet the measurements were markedly higher than the same locations in the canine cornea. Lastly, the mean AFM measurements of the anterior and posterior stroma from the dog were similar to AFM measurements previously reported in the rabbit but were less than those determined for the human (Table 1) [8]. While quantitative differences were found between species, overall, the anterior stroma was consistently stiffer than the posterior stroma irrespective of species examined (dog, rabbit, human).

## 4. Discussion

This study defined the collagen stromal arrangement of the canine cornea and correlated this pattern of organization with stromal stiffness. A higher degree of collagen fiber intertwining was identified in the anterior stroma compared with a more lamellar arrangement in the posterior stroma. The measurements of stromal stiffness were significantly higher in the anterior stroma and lower in the posterior stroma, in both the dog and human cornea. This pattern of collagen stromal arrangement, and subsequent stromal stiffness is a conserved feature seen in other mammals that have been examined to date [6,8,14]. The conservation of both the structural and biomechanical properties amongst other mammals, including humans, reflects a conserved evolutionary pressure for maintaining corneal strength and corneal curvature of the anterior stroma for refraction and vision.

The use of SHG imaging has greatly enhanced the ability to examine collagen fiber arrangement with the stroma of the cornea. This was recently demonstrated in a comparative study of corneal collagen organization that uncovered a wide variation in collagen fiber organization across the five classes of vertebrates (fish-great white shark, amphibians-bullfrog, reptiles-alligator, birds-falcon and mammals-human) [7]. Interestingly, the non-mammalian species had a simpler lamellar organization with very little branching in the lower vertebrates (fish, amphibians and reptiles), and with increased branching being observed in higher vertebrates (mammals and birds). Mammalian corneas possess spatially distinct structural organization with intertwining of the collagen fibers in the anterior stroma and a more parallel arrangement in the posterior cornea [7]. Differentiating the mammalian corneal collagen arrangement was the degree of intertwining in the anterior corneal stroma and the depth at which it transitions to a more parallel organization with no intertwining [8]. This is certainly evident when comparing the collagen fiber intertwining of the canine and rabbit cornea with that of humans. It has been shown in rabbits, and now dogs, that the area of intertwining is limited to the anterior 30% of the corneal stroma, whereas the human extends throughout the anterior 80% of the stroma [6,8].

The consistent finding of a high degree of intertwining in the anterior corneal stroma amongst mammals suggests that there is a significant evolutionary pressure to maintain this characteristic of the cornea for rigidity, curvature and light refraction. The relationship between the three-dimensional organization of the collagen fibers and the biophysical attributes of the cornea suggests that the mechanobiology of the cornea would be impacted by certain corneal pathologies and surgical interventions. For example, canine patients that undergo superficial keratectomy for superficial corneal pathology or spontaneous chronic corneal epithelial defects (SCCEDs) would result in extirpation of this important area of intertwined collagen fibers. These animals would likely have permanent alteration in the rigidity of the anterior stroma. This is in addition to induced altered corneal curvature, light refraction and vision, features essential for service animals and others requiring high visual acuity.

There is a clinical need to develop synthetic and bioartificial corneas for use in transplantation in patients with corneal pathology; therefore, the development of models to study transplant physiology and serve as a source of xenograft material is critically important. However, if there are key biomechanical differences between seemingly identical tissue at the light microscopy level, these differences may represent significant complications in the utilization of xenograft material. The data presented in this study demonstrate that, despite the overall similarity in collagen arrangement between humans and dogs, the different percentage of collagen fiber intertwining in the cornea leads to important variations in tissue stiffness. To more thoroughly investigate the structural differences that lead to variations in tissue biomechanics, further investigations are required that focus on (1) quantification of collagen fiber intertwining; (2) definition of glycosaminoglycans and proteoglycans composition that bridge elastic and collagen fibers; (3) determination of corneal ultrastructure with serial block face scanning transmission electron microscopy (EM) or cryopreserved EM; and (4) the organization of the stromal matrix using x-ray scattering or multi-photon techniques. When viewed from the clinical application of transplantation, the reduced tissue stiffness in the anterior stroma of non-human mammals may be a significant impediment to transplantation into humans due to alterations in corneal curvature and refraction. Also, species-based considerations may be essential when evaluating the safety and efficacy of responses to drugs for treating corneal disorders. Therefore, the design and generation of biosynthetic and bioartificial corneal tissue will likely be highly influenced by the collagen organization and biomechanical attributes.

The values obtained for tissue elastic modulus of a biologic tissue can vary dramatically depending on the specific method used (tensile vs. indention) [13]. Even when restricted, by using the indentation method of AFM, values can vary depending on the tip size, scan rates and indentation depths. For example, the mean elastic modulus of the anterior corneal stroma of the human has been reported to be 1.4 kPa, 33.1 kPa, or in a range from 1.14–2.63 MPa, and this discrepancy is largely dependent on the technique (e.g., indentation vs. tensile stretching) and specific details (e.g., tip size, depth) of the instrumentation used [6,15,16].

Overall, this study determined that the organization of the canine corneal stroma is similar to other mammalian species and is characterized by the presence of intertwining of the collagen in the anterior stroma. Tissue stiffness directly correlated with the degree of intertwining of the stromal fibers, with the anterior stroma being stiffer. These studies provide a touchstone for future studies evaluating the consequence of corneal pathologies and surgical interventions on the mechanobiology of the cornea.

## Figures and Tables

**Table 1:** The tissue stiffness of the anterior and posterior corneal stroma is similar between dogs, rabbits and humans. Atomic force microscopy (AFM) measurements of the anterior and posterior corneal stroma expressed as mean kilopascals (kPa) ± standard deviation (SD).

Species	Anterior Stroma	Posterior Stroma
Rabbit [8]	1.1 ± 0.6	0.4 ± 0.2
Canine	1.3 ± 1.0	0.5 ± 0.3
Human	16.2 ± 2.5	2.5 ± 1.5
